# Long-term associations among male sperm whales (*Physeter macrocephalus*)

**DOI:** 10.1371/journal.pone.0244204

**Published:** 2020-12-23

**Authors:** Hayao Kobayashi, Hal Whitehead, Masao Amano

**Affiliations:** 1 Graduate School of Fisheries and Environmental Science, Nagasaki University, Nagasaki, Japan; 2 Department of Biology, Dalhousie University, Halifax, NS, Canada; Hawaii Pacific University, UNITED STATES

## Abstract

Little is known about the social structure of male sperm whales (Physeter macrocephalus) after they leave their natal units. While previous studies found no evidence for preferred associations among males, the observation of mass-strandings consisting exclusively of males, suggest that they have strong social bonds. To investigate the social associations among male sperm whales, we used half weight index of association, permutation tests and standardized lagged association rate models on a large photo-identification database collected between 2006 and 2017 in Nemuro Strait, Japan. Our results suggest that while male sperm whales are not as social as females, they do form long term associations, have preferred companionship, and forage in social proximity to each other. The best-fitting model to the standardized lagged association rate showed that associations among males last for at least 2.7 years and as most males leave the area after 2 years, associations may last for longer. Twenty dyads were observed associating over more than 2 years, for a maximum 5 years. One dyad was observed associating on 19 different days and clustered on 7 different days. Male associations may function to enhance foraging or to fend off predators. Such relationships seem to be adapted to a pelagic habitat with uncertain resource availability and predation pressure.

## Introduction

Among mammals, male relationships tend to be competitive [1], and consistently strong association rates are relatively rare. In some species, males form strong relationships to defend estrous females, or access to female groups or territory (e.g., chimpanzee *Pan troglodytes*: [2]; bottlenose dolphin *Tursiops* sp.: [3]; lion *Panthera leo*: [4]; cheetah *Acinonyx jubatus*: [5]). Most of these associated males are kin. Although male sperm whales, *Physeter macrocephalus*, form all-male groups, the factors promoting group formation among males may be quite different from those in species where males associate with related males to improve mating success.

Female sperm whales and their offspring live in stable social units at low or mid-latitudes [6], and most females remain within their natal unit throughout their life [7]. In contrast, males leave their natal unit before sexual maturity (~6–16 years) and are described as forming “bachelor schools” consisting of males of about the same age, generally at high latitudes outside the females’ range [8]. The sizes of bachelor schools have a negative correlation with mean body length within the school suggesting diminishing sociality with age [8–10]. Finally, males, in their forties and older, may be seen singly in high latitude areas including near the ice edges, as well as in lower latitude areas where they migrate to mate [6, 11]. Therefore, from what we know, male relationships within bachelor schools of sperm whales are quite different from those in alliances or coalitions which relate to defense of females or territory as no females are present, or even nearby, at these higher latitude. However, little is known about the extent of sociality in bachelor schools.

Mass stranding events–when two or more animals beach themselves at the same place and time- suggest that male sperm whales can form cohesive male groups. Mass strandings are commonly reported for highly social odontocetes such as short-finned pilot whales *Globicephala macrorhynchus*, long-finned pilot whales *Globicephala melas* and false killer whales *Pseudorca crassidens* [12]. Sperm whales sometimes also mass strand, and some mass stranding consist only of males [6]. For instance, Rice [13] reported a total of thirteen all-male strandings including 3 to 37 animals (mean 12.5 animals). These events suggest that there may be strong associations among males. However, there is no information about their associations before stranding. Bond [14] found that among two all-male mass strandings on the coast of Scotland, most animals were genetically unrelated to one another. Autenrieth et al.’s [15] genetic analysis suggested that the 27 male sperm whales that stranded together in the North Sea were not maternally related individuals but instead included assemblages of individuals from different natal geographic regions. Schnitzler et al. [16] also reported evidence for at least two cohorts with different origins among 24 of these males based on contaminant and genetic analyses. Considering the long calving intervals of individual females (every 4~6 years) [13, 17] and the size of female family units (~10; [6]), it would be almost impossible to form an all-male group consisting of more than very few related males of approximately the same age. Taken together, these results suggest factors other than kinship structure male relationships.

Despite observations of “bachelor schools” and mass strandings of nonbreeding males [9], there is little evidence from modern studies of living sperm whales that males form groups with preferred association [6]. In visual and acoustic surveys at high latitudes, aggregations of males spanning from 10 to 30 km are commonly found [e.g., 18, 19]. Such aggregations were also described by whalers [20]. Christal and Whitehead [21] found male sperm whales swimming alone within aggregations of males spreading over 20 km across off the Galapagos Islands. Members of the male aggregations usually showed consistent heading mostly within about 20° of the modal heading of the aggregation on the same day, but interactions between members were not observed [21]. Therefore, it is unclear whether the males’ coordinated movements were a response to external factors such as the distribution of prey or geographical features such as the presence of canyons, or reflected social bonds between the males themselves.

Lettevall et al. [22] reported no evidence of preferred companionship or long-term relationships among photo-identified males from 4 study areas: Andenes (Norway); The Gully (Nova Scotia, Canada); off Galapagos Islands (Ecuador); Kaikoura (New Zealand). Thus, among males, there is no evidence of the long-term relationships, or social units, that are characteristic of females and their dependent young. However, in these studies, identification data were fairly sparse and perhaps insufficient to detect some forms of long-term social structure. Moreover, Lettevall et al. [22] also mentioned the possibility of more social groups consisting of relatively small and young males based on heading coordination within aggregations and mass strandings. Thus, the social relationships between male sperm whales are still poorly known.

Here, we try to fill this gap in knowledge by investigation the social associations among nonbreeding male sperm whales in the Nemuro Strait, Hokkaido, Japan. This area is a summer feeding ground for male sperm whales and has been used extensively for commercial whale watching during which photo-identification has been conducted since 2006. We used this photo-identification dataset to characterize the nature of male relationships and show that males have consistent long-term associations.

## Materials & methods

The study complied with the laws of Japan and was performed in accordance with the Guidelines for Animal Experimentation of Nagasaki University with approval of the Institutional Animal Care and Use Committee.

### Field methods

**Fig 1:**
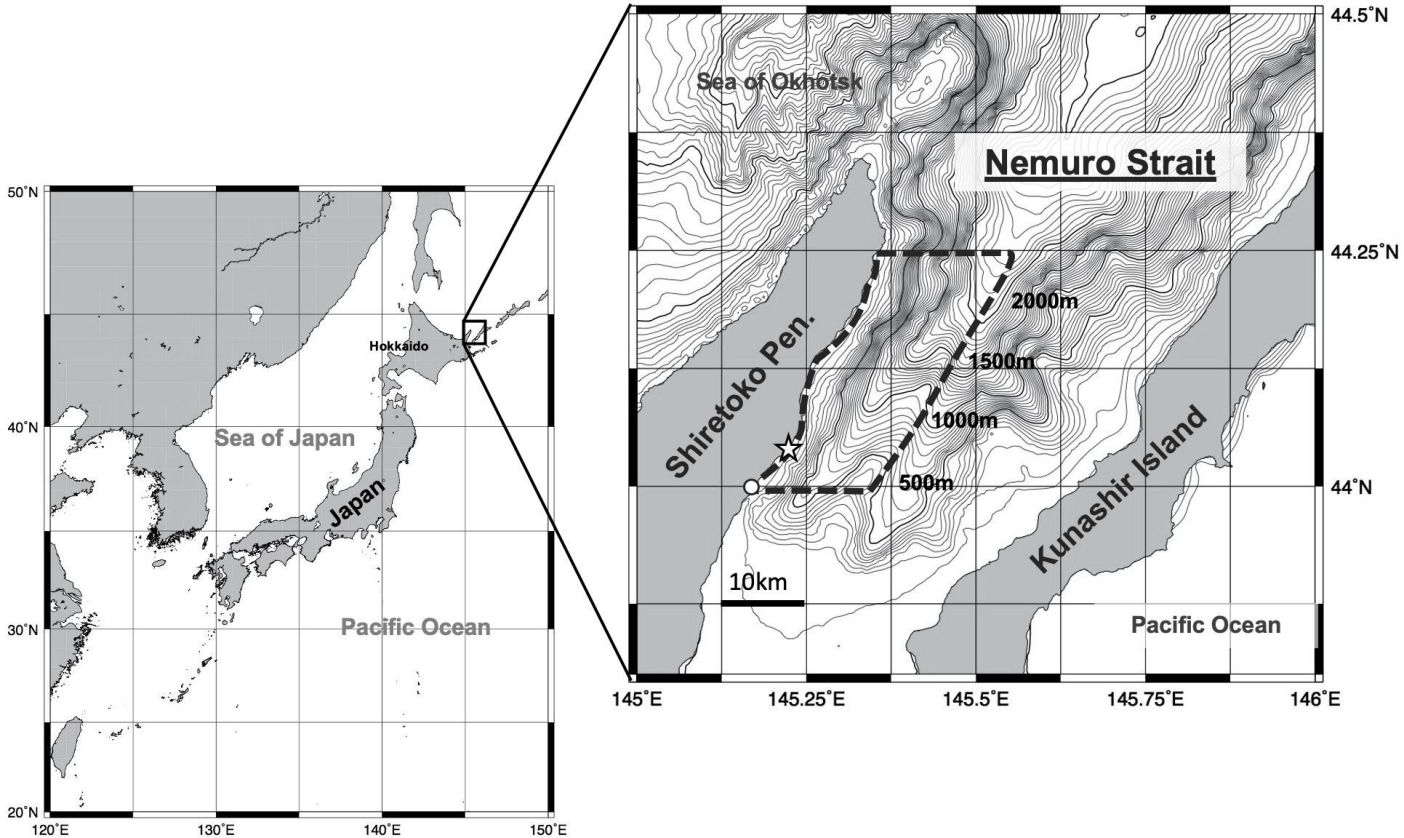
Map of the location of Nemuro Strait, Hokkaido, Japan. Broken line shows the range of study area, and open circles indicates position of “Rausu Fishing Port” (departure point of whale-watching boat). Open star shows the “Whale View Park” (theodolite station, see S1 Fig). The maps were created by using GMT: The Generic Mapping Tools (ver. 5.4.5; https://www.generic-mapping-tools.org/, Wessel et al. 2013 [55]), and the depth contours were created on GMT based on “500m Gridded Bathymetry Data" provided by Japan Coast Guard (Japan Oceanographic Data Center (JODC) 500m Gridded Bathymetry Data: https://jdoss1.jodc.go.jp/vpage/depth500_file.html).

Field work was conducted in Nemuro Strait (43° 58’ N, 145° 10’– 145° 15’ E, 44° 20’ N, 145° 23’– 145° 35’ E, Fig 1) during summer field seasons (mainly between July and September) over 12 years (from 2006 to 2017, a total of 890 days). Photo-identification data were collected from 5 commercial whale-watching boats in the Strait. Each cruise lasted approximately 2.5 hours and most boats operated twice a day. During the surveys, we searched for whales visually using binoculars and acoustically using hydrophones. Whale watching vessels shared information on whale detections with each other. Although the survey route differed slightly from day to day (depending on the weather and sea surface conditions or on the presence of whales), whales were mostly found some 5–15 nautical miles (nmi) from the fishing port of Rausu where the water depth is between 500–1,500 m deep (Fig 1).

### Individual identification

Sperm whales, as well as many other cetacean species, can be identified individually based the shape, coloration and marks on their flukes or dorsal fins [23]. In previous sperm whale studies, 90% of encountered individual could be reliably identified this way [24]. Photographs of the flukes of diving sperm whales were taken with an APS-C digital single-lens reflex camera (Canon EOS 40D, 70D, 7D, and Nikon D7000) equipped with a 70–300 mm zoom lens. Photographs were matched to an identification catalog following the methods of Arnbom [25]. A quality rating (Q) between 1 and 5 was assigned to each photograph and only high-quality photographs with a Q ≧ 4 were used for the analyses. Photo-identification data were not collected blindly (i.e., we often knew the identities of the animals that we were photographing) because our study involved observations of focal animals in the field.

### Data analysis

In the framework proposed by Hinde [26], the social structure of a population is a synthesis of the pattern of the relationships among its members, which in turn are described by the nature and quality of their interactions [11, 27]. In most cetacean species, social interactions occur under the sea surface which makes them difficult to quantify. Because of this, many researchers studying social structure in cetaceans make an assumption called “the gambit of the group”: individuals are assumed to be interacting if they are found in the same location at the same time [28]. Thus, the relationships between pairs of individuals can be described by the characteristics and temporal patterning of their associations [26, 27, 29].

Previous sperm whale studies have quantified associations two ways: either spatially by assuming that individuals within a cluster (within 3 body lengths of each other and coordinating movements) are associating (e.g., [30–32]) or temporally by assuming that sperm whales identified within 10 min (e.g., [33]), 2 hour (e.g., [22, 34–36] or in same day (e.g., [22, 36]) are associating. Whales in clusters are assumed to be associating since they are in visual, and sometimes physical, contact with each other while whales encountered within 2 hours are assumed to be associating since they are in acoustic contact with each other (the audible range through hydrophones is 16 km for “usual clicks” (searching echolocation clicks) and 60 km for “slow clicks” (clicks used by males) [6 data from 37]. Previous studies (e.g., [7, 32]) also showed that using stronger measures of association (such as “in a cluster together” and 10 min criterion), as compared to those with looser criteria, did not affect the outcome of permutation tests for preferred associations.

**Fig 2:**
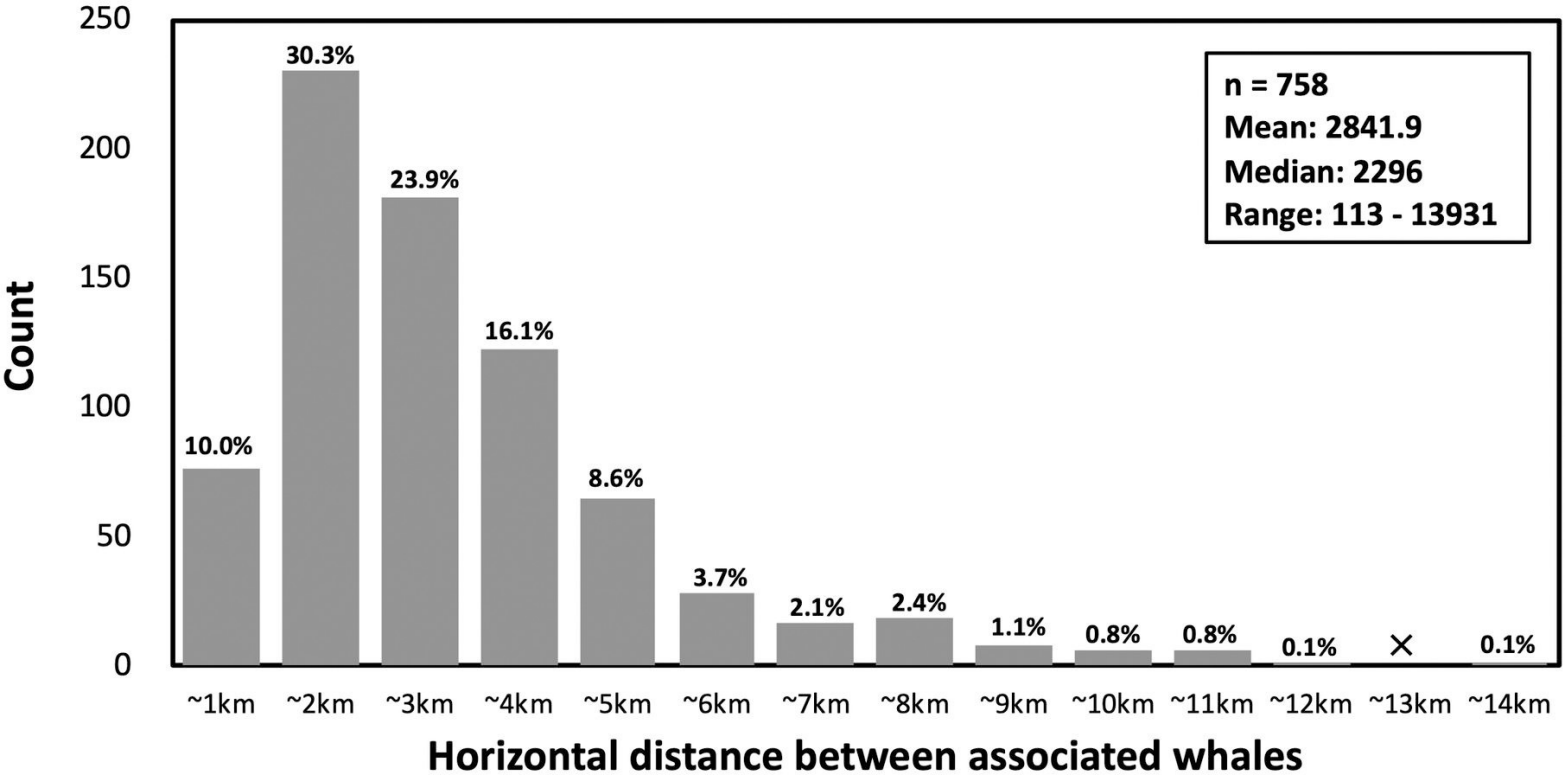
Distribution of horizontal distance between locations of observations of associating whales (identified within 1 hour) from the research vessel.

In this study, we considered whales identified within 1 hour of each other from the same boat to be associated. Associated whales were observed 2,841 ± 1,961 m (mean ± SD) apart on average (n = 758, Fig 2). In the Strait, sperm whales dispersed over wider ranges: in land-based visual surveys an average of 5.5 animals were identified per hour, with an average horizontal distance of 5,778 ± 3,786 m (mean ± SD, n = 9,790; S1 Fig) between them. This distance is twice as wide as that between associated animals identified from the research vessels. Thus, associating whales (sighted within 1 hour) are proximate animals in the foraging area, and are likely to be within acoustic contact of each other.

To quantify the strength of associations between individuals, we used the half-weight index (HWI). An HWI equal to zero indicates that the dyad never associated -as per our definition of association- and an HWI equal to one indicates that the members of the dyad were always observed within 1 hour of each other [27]. The sampling period (unit of analysis) was 1 day, so that the association dataset indicates which individuals were associating on which days.

### Preferred associations

A permutation test examined the null hypothesis that associations between individual males were random given the temporal pattern of each individual’s identifications. In this test, we permuted associations within samples. The association matrices for each day were randomized 10,000 times with 10,000 flips per permutation maintaining the number of associates of each individual on each day, with HWIs being calculated after each permutation, at which point the P-values stabilized [37]. If some pairs of animals were preferentially associating with or avoiding one another over different days, then this would increase variation among the HWIs. Thus, if the standard deviation or the coefficient of variation of the observed association indices was significantly higher than those calculated from the randomly permuted data, the null hypothesis (no preferential associates over days) was rejected [27].

### Temporal pattern

Temporal change in association was examined by calculating the standardized lagged association rates (SLAR) for associations with identification data from 225 individuals. The standardized lagged association rate is an estimate of the probability that if two individuals are associated at any time, then, after a given time lag, the second individual will be a randomly chosen associate of the first [29]. The SLAR was compared to the standardized null association rates (SNAR): that is the expected SLAR if individuals associated at random. Four exponential models were fitted to SLAR to describe the temporal patterning of male associations in Nemuro Strait: the first model had no decay and suggests permanent associations; the second model had a decay down to zero and suggests that associations decrease until complete disassociation; the third model had a decay that levelled off and suggesting both long-lasting and temporary associations; and the fourth model had two decays and suggested two levels of disassociation, at a shorter and longer time lags respectively [27]. The standard error of the SLAR was estimated using jackknife methods [27]. The best fitting model was chosen based on the lowest Quasi Akaike Information Criterion (QAIC). All social analyses were carried out in the SOCPROG 2.8. software [38].

### Cluster

Clusters are sets of sperm whales observed at the surface within 3 body lengths of one another and swimming in the same direction [39]. During social or resting periods, it is typical for females and immatures to cluster [6]. Therefore, clusters can be regarded as a signal of social interaction among sperm whales. In contrast to females, most nonbreeding males are seen alone at the surface [22], however, these males do sometimes actively cluster [20]. Thus, when we encountered a cluster, we recorded the cluster size, the behavioral mode (surfacing between dives, socializing, resting), and identified the individual members of the cluster whenever possible.

## Results

We obtained 2,968 identifications of 226 individual male sperm whales on 608 different days in the Nemuro Strait. 44 photographs (out of a total of 3012, 1.5%) could not be identified due to the absence of marking on the sperm whales’ flukes. The discovery curve for individuals is still increasing steadily as new individuals are identified every year (S2 Fig). 127 individuals (56.2%) were identified over multiple years (2–9 times). Individuals were identified on a mean of 14 days each, and on each day had a mean of 1.4 associates (identified within 1 hour). The mean association index was 0.130. The mean cluster size (including single animal clusters) in Nemuro Strait was 1.07 individuals (cluster size range: 1–7), so that males were usually seen alone.

The standard deviation and the coefficient of variation of the association indices were significantly higher for the observed than for the randomized data (P < 0.01, Table 1). Thus, the null hypothesis that there are no preferential associations among males between sampling periods (days) was rejected.

**Table 1:** Permutation test for preferential association between single day periods.

	Observed data	Random data	P-value
SD of mean association index	0.03248	0.03134	0.0071
CV of mean association index	8.69431	8.42104	0.0002

**Fig 3:**
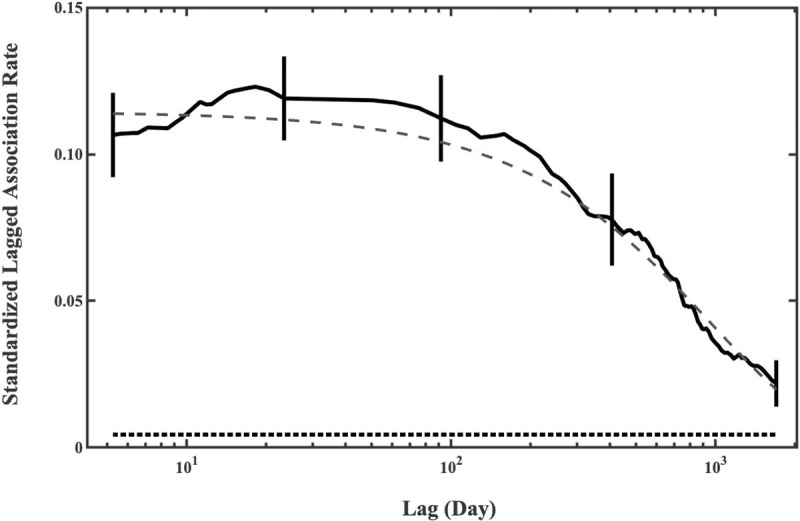
Standardized lagged association rate for males in the Nemuro Strait, with jack-knifed estimates of precision. The SLAR (black line), SNAR (dot line; the theoretical SLAR if the individuals are randomly associated) and best-fitting model (broken line) are shown for the males in Nemuro Strait.

The SLAR was higher than the SNAR for time lags less than approximately 1700 days (Fig 3). Therefore, some pairs of individuals were more often associated than expected if association was random for periods of up to about 5 years. The SLAR was highest for short time lags, and decreased after approximately 100 days, indicating that many associations between individuals last for at least 3 months, the approximate duration of a field season. A model with a simple term of exponential decay in relationship strength best fitted the SLAR data (Table 2). The mean duration of association estimated from the best model is 968 days (inverse of exponential parameter; Table 2).

**Table 2:** Exponential decay models fitted to the standardized lagged association rate among male sperm whales in the Nemuro Strait.

Model‘s Explanation	Fitted Model	QAIC	ΔQAIC
Preferred companions	0.07	10782.68	443.37
**Casual acquaintances**	10782.6827*exp(-0.0010329*td)	**10339.31**	**0**
Constant companions and casual acquaintances	-0.00044404+0.11483*exp(-0.0010235*td)	10341.31	2.00
Two levels of casual acquaintance	-0.0068166*exp(-0.013092*td)+0.11925*exp(-0.0010765*td)	10342.90	3.59

The lowest Quasi Akaike Information Criterion value (QAIC) indicates the best-fitted model. ΔQAIC is the difference between the QAIC for the current model and best-fitted model. Duration of association estimated from the best model is 968 days.

**Fig 4:**
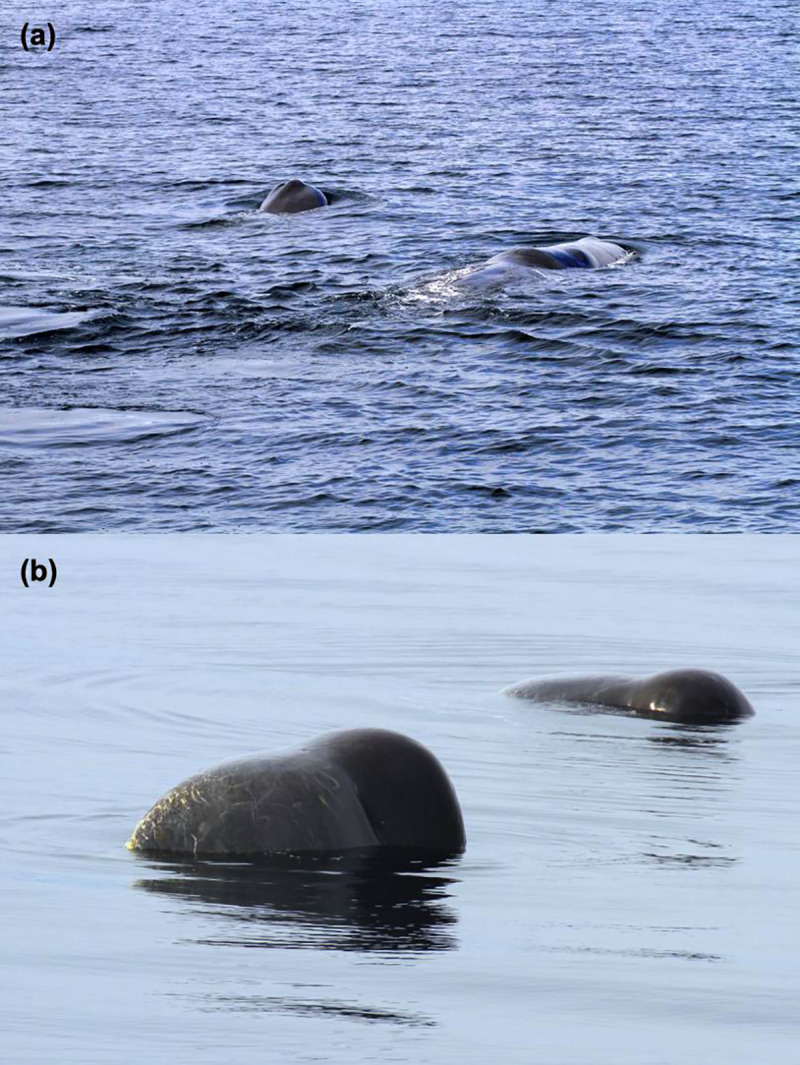
Two resting male sperm whales (NS-PM099 and NS-PM101) photographed on (a) August 27 and (b) September 13, 2013.

Twenty dyads were observed associated over more than two years (Table 3). The individual males NS-PM101 and NS-PM118 have been observed associated on 29 days over 4 different years (2011, 2013, 2015, 2016). For 55 clusters with two or more individuals, we could identify all the individuals composing the cluster. Forty-one clustered pairs were identified, 6 of which formed a cluster more than once. These pairs were observed association over more than 2 years, and 5 of the 6 pairs observed in the same cluster multiple times had at least twice the mean association index of all dyads. The individuals NS-PM099 and NS-PM101 were observed associated on 19 different days in 3 different years and were observed in clusters together on 7 different days. On two of those occasions, August 27 and September 13, 2013, the whales NS-PM099 and NS-PM101 rubbed and rested together at the surface (Fig 4).

**Table 3:** Twenty dyads observed associated during more than 2 years.

Dyads		Year	No. of association	HWI	No. of clustering
NS-PM089	NS-PM090	2008, 2010, 2011, 2012, 2014	10	0.15	0
NS-PM101	NS-PM118	**2011**, 2013, **2015**, **2016**	29	0.35	3
NS-PM082	NS-PM083	**2008**, 2009, **2011**, 2013	10	0.26	3
NS-PM082	NS-PM090	2009, 2011, 2012, 2014	8	0.12	0
NS-PM082	NS-PM132	2011, 2012, 2013, 2014	5	0.12	0
NS-PM090	NS-PM132	2010, 2011, 2012, 2014	5	0.11	0
NS-PM031	NS-PM033	2006, 2007, 2011, 2014	4	0.15	0
NS-PM099	NS-PM101	2009, **2011**, **2013**	19	0.29	7
NS-PM101	NS-PM163	2013, 2015, 2016	10	0.16	0
NS-PM167	NS-PM169	2014, 2015, 2016	10	0.32	0
NS-PM118	NS-PM163	2013, 2015, 2016	9	0.17	0
NS-PM099	NS-PM118	**2009**, **2011**, 2013	6	0.11	2
NS-PM059	NS-PM063	2007, 2009, 2012	5	0.25	0
NS-PM033	NS-PM059	2007, 2009, 2010	4	0.13	0
NS-PM006	NS-PM007	**2006**, 2007, 2013	4	0.29	1
NS-PM089	NS-PM119	2010, 2012, 2014	4	0.11	0
NS-PM089	NS-PM132	2010, 2012, 2014	4	0.10	0
NS-PM082	NS-PM092	2008, 2009, 2011	3	0.09	0
NS-PM083	NS-PM092	2008, 2009, 2011	3	0.30	0
NS-PM090	NS-PM096	2010, 2011, 2014	3	0.06	0

Bold shows years which the dyads were observed in the same cluster.

## Discussion

### Long-term associations among male sperm whales

Our study suggests that male sperm whales can form long-term associations. We note, however, that associations between male sperm whales in the Nemuro Strait (mean index = 0.130) are much lower than those within female social units (e.g., off the Galapagos Islands the mean index is 0.399; [33]). This is despite the fact that the Galapagos study used a tighter definition of association for females (identified within 10min [33]). Our findings strongly reinforce conclusions from previous studies that males are less social than females (e.g., [21, 22]).

On the other hand, our results also suggest that, although low, associations among some males in Nemuro Strait are not random. Male sperm whales are feeding relatively close to their preferred associates within the foraging ground where multiple males are dispersed. Letteval et al. [22] found that association patterns of male sperm whales in 4 research areas were not significantly different from random. However, this does not necessarily mean that males in these study areas are not social. Considerable identification data are needed to detect preferred companionships [40], especially when the rate of associations is low. Therefore, the apparent discrepancy between our results and those of Letteval et al. [22] may reflect the smaller dataset of the earlier study. Differences between sperm whales of different regions could also play a part. Whitehead et al. [30] revealed a clear contrast in the social structures of female sperm whales between the eastern Pacific and North Atlantic Oceans, likely due to differences in predation pressure between the ocean basins [30]. In addition, further investigation is needed to reveal whether the social structure varies geographically in males as well.

We also found that the male sperm whales associate for about 2.7 years on average. The timing and pattern of decline in their rates of association is similar to that of the lagged identification rate (probability that an animal is still in a study area after different time lags; [27, 41]) which indicated that the males stay for a mean of 2.1 years in the Strait [42]. This suggests that the decline of association rates is unlikely to be caused only by dis-association among dyads, but instead is primarily caused by death or emigration to other areas by one member of the pairs. Therefore, preferred relationships among males could last for longer periods, more than 2.7 years, perhaps for 5 years, considering our findings that the empirical association rates were higher than the null expectancy for lags of about 5 years. Taken together, these results suggest that while male sperm whales are not as social as females, they have long-term relationships, preferred associations and forage in close spatial proximity.

In mid and high-latitude areas, males make repeated foraging dives lasting about 40 minutes, separated by about 7 minutes breathing at the surface (e.g., [35, 43, 44]). During those foraging dives, the usual echolocation clicks and slow clicks (thought to be communicative vocalizations of males) are produced (e.g., [44, 45]). Madsen and Møhl [45] estimated that sperm whales may be able to hear each other at ranges of 16 km for usual clicks, and up to 60 km for slow clicks. Thus, pairs of males deemed to be associated were within the presumed audible range of echolocation clicks, and males might obtain information about prey distribution or feeding success from the echolocation clicks of others. Synchrony of horizontal movement among males in Nemuro Strait (Amano & Kobayashi, personal observation) and heading coordination within aggregations of males as reported by Christal and Whitehead [21] may also be caused by such interaction among neighboring males while foraging. Christal and Whitehead [33] suggested that a possible value of long-term relationship among females is communal sharing of information about resources of high uncertainty over their large home ranges. A similar function of “ecological enhancement” may also promote the long-term social relationships among males. However, since there are few observations of direct interactions during foraging dives between males, further study is needed to test this hypothesis.

Although pairs of male sperm whales which preferentially associate for long periods tend to be observed in close spatial proximity, most individual whales identified in Nemuro Strait are kilometers apart from one other. Males within long-term preferred social relationships sometimes gather and rest with their companions at the surface as females do. The association indices of pairs displaying such relationships tended to be higher. However, this cannot be rigorously statistically tested because the data are limited and there is a structural correlation between association index and frequency of clustering. Nevertheless, the data on identifications within clusters identified, while sparse, provide further evidence of social relationships between males across years.

Previous studies of male sociality from 4 different areas reported males usually alone at the surface with few clusters of 2 or more whales. Compared to our study, repeated resighting of clusters consisting of the same pair of individuals was much rarer and the interval of observations between these repeat clusters were quite short-term: from several tens of minutes to 3 days [22]. Although further research is needed to explain the discrepancies between our results and these, one possible explanation for the low frequency of cluster resights is that whales clustered at the surface are often hard to identify since they often do not fluke up following social interactions as they do during foraging dives.

Female sperm whales form clusters near the surface almost every day to socialize and rest with members of their social units and with others within a larger, temporary group [34, 46]. These behaviors may be important for maintaining the social bonds between members of a social unit after dispersion during foraging dives [34]. Though the frequency of forming clusters is lower than among females, males may also form clusters to maintain social bonds, suggesting that social life is still significant for males.

Cluster formation by males may also have a function as anti-predation behavior. Curé et al. [47] reported that large males over 15 m long interrupted their foraging or resting dives and formed clusters with other males or produced coda vocalizations in response to playbacks of vocalizations from their major natural predator, the killer whale, Orcinus orca [6]. Codas play a major role in communication among sperm whales, but codas have only rarely been recorded in the high latitude habitats of males [44, 45, 48]. These experimental results suggest that large males are not completely solitary and interact with neighboring males when threatened by predators. Cluster formation by resting whales may also have an anti-predator function [6]. Hence, predation pressure, probably by killer whales, may play an important role in promoting social relationships between males. In addition, cohesive bachelor schools observed by whalers and scientists during the modern whaling period (e.g., [8–10]) might have a similar root, with animals responding to the presence of whaling ships. In recent centuries, the most dangerous predators for sperm whales have been humans not killer whales. At sperm whaling’s peak, over 20,000 animals per year were caught globally (from [49]), and about 2,000 animals were also killed every year by Japanese coastal whaling during the late 1960s [50]. Although many scientists (e.g., [8–10]) and whalers described cohesive “bachelor schools” of male sperm whales, there have been few observations of such groupings during studies of living animals following the whaling period [22]. Hunting has likely affected not only sperm whale populations but also their behavior.

### Long-term associations in male mammals

Our study found social relationships among non-breeding male sperm whales and suggests that they may be important. Long-term relationships between non-reproductive (and generally unrelated) males are rare among mammals. Often, in non-solitary mammalian species, reproductive-age males live with females, either as monogamous pairs (e.g., prosimians, gibbons), as members of well-structured closed groups (e.g., most primates, lions, horses), or larger, looser, more promiscuous groupings (e.g., some larger ungulates, dolphins) [51]. The males within groups may have relationships, sometimes strong and important relationships, but they are thought to be based on the enhancement of reproductive success (e.g., chimpanzees; [2]). When males live largely apart from females, there may also form strong bonds which function during breeding attempts, for instance as alliances (e.g., bottlenose dolphins; [3]). However, strong relationships among non-breeding males without females being present or in prospect seem rare especially when males are unrelated.

A possible exception is the African elephant (*Loxodonta africana*) which has a similar life history to the sperm whale (see [8, 52]), although the motivation of association between males may be much different from that of sperm whales. After leaving their maternal units, male elephants tend to associate with males about the same age sometimes acting as sparring partners, or with older bulls who may be reservoirs of social and ecological knowledge within breeding herds [53]. Male elephants’ associations are positively correlated with genetic relatedness [54] unlike sperm whales which associate with unrelated males of the same size. These difference show that associations among non-breeding male elephants may more directly function in increasing mating success than is the case with sperm whales where geographical segregation between the sexes is much more extreme (1,000s km for sperm whales; 10s km for elephants).

### Conclusion

Beyond mammals, we know of no evidence of long-term bonds between unrelated non-breeding males in birds, reptiles or amphibians. Some instances may have been missed by scientific studies, or by us when surveying the literature. However, such cases are almost certainly very rare. Sperm whales seem to be an unusual species in which male bonds are not based on reproduction or kinship. This sociality may be promoted by the importance of cooperation in a pelagic habitat (likely for cooperative foraging or anti-predation) and the extreme spatial separation of the two sexes for prolonged periods in a species which is otherwise highly social. To understand the social structure of male sperm whales more deeply, relationships between male sperm whales should be examined for longer periods of time, in additional study sites, over a wider range of ages, and in more behavioral detail.

## Supporting information

S1 Fig The distribution of horizontal distance between whales observed within 1 hour from land-based survey. Surveys were carried out from the Whale View Park in Rausu Town, Hokkaido, Japan (44°02'N, 145°13'E; 73 m above sea level) on 256 days from 2010 to 2019. Observation range is about 15 nmi, which substantially coincides with the range of photo-identification research. Four observers searched for whales using binoculars (Nikon MONARCH 12×42) and the position of each whale was recorded using digital theodolite (SOKKIA DT5). The distances between whales identified within 1 hour were calculated using the data collected during the 139 days for which the visibility was over 10 nmi and Beaufort Wind Scale was less than 5).
(DOCX)

S2 Fig Discovery curves for individuals identified in Nermuro Strait between 2006 and 2017. (DOCX)

S1 File Data used for all analyses. (XLSX)

## References

[1] van Hooff JA, van Schaik CP (1994) Male bonds: afilliative relationships among nonhuman primate males. Behaviour 130:309–337. 10.1163/156853994X00587

[2] Mitani JC, Merriwether DA, Zhang C (2000) Male affiliation, cooperation and kinship in wild chimpanzees. Anim Behav 59:885–893. 10.1006/anbe.1999.1389 10792944

[3] Connor RC, Smolker RA, Richrds AF (1992) Two levels of alliance formation among male bottlenose dolphins (*Tursiops* sp.). Proc. Natl Acad Sci 89:987–990. 10.1073/pnas.89.3.987 11607275 PMC48370

[4] Packer C, Pusey AE (1982) Cooperation and competition within coalitions of male lions: Kin selection or game theory? Nature 296:740 10.1038/296740a0

[5] Caro TM, Collins DA (1987) Male cheetah social organization and territoriality. Ethology 74:52–64. 10.1111/j.1439-0310.1987.tb00921.x

[6] Whitehead H (2003) Sperm whales: social evolution in the ocean. University of Chicago Press, Chicago.

[7] Christal J, Whitehead H, Lettevall E (1998) Sperm whale social units: variation and change. Can J Zool 76:1431–1440. 10.1139/z98-087

[8] Best PB (1979) Social organization in sperm whales, *Physeter macrocephalus* In: Winn HE, Olla BL (eds) Behaviour of marine animals: cetaceans. vol. 3 Plenum, New York, pp 227–289

[9] Gaskin DE (1970) Composition of schools of sperm whales *Physeter catodon* Linn. east of New Zealand. N Z J Mar Freshw Res 4:456–471. 10.1080/00288330.1970.9515359

[10] Ohsumi S (1971) Some investigations on the school structure of sperm whale. Sci Rep Whales Res Inst 23:1–25

[11] Whitehead H, Weilgart L (2000) The sperm whale: Social females and roving males In: Mann J, Connor RC, Tyack P, Whitehead H (eds) Cetacena societies, University of Chicago Press, Chicago, pp 154–172

[12] Sergeant DE (1982) Mass strandings of toothed whales (Odontoceti) as a population phenomenon. Sci Rep Whales Res Inst 34:1–47

[13] Rice DW (1989) Sperm whale *Physeter macrocephalus* Linneaus, 1758 In: Ridgway SH, Harrison RJ (eds) Handbook of marine mammals, Vol 4 Academic Press, London, pp 177–233

[14] Bond J (1999) Genetic analysis of the sperm whale (*Physeter macrocephalus*) using microsatellites. PhD Dissertation, University of Cambridge, Cambridge.

[15] Autenrieth M, Ernst A, Deaville R, Demaret F, IJsseldijk LL, Siebert U, et al (2018). Putative origin and maternal relatedness of male sperm whales (*Physeter macrocephalus*) recently stranded in the North Sea. Mamm Biol 88:156–160. 10.1016/j.mambio.2017.09.003

[16] Schnitzler JG, Pinzone M, Autenrieth M, van Neer A, IJsseldijk LL, Barber JL, et al (2018) Inter-individual differences in contamination profiles as tracer of social group association in stranded Sperm whales. Sci Rep 8:10958 10.1038/s41598-018-29186-z 30026609 PMC6053436

[17] Best PB, Canham PAS, Macleod N (1984) Patterns of reproduction in sperm whales, Physeter macrocephalus. Rep Int Whal Comm. (Special Issue) 6:51–79

[18] Gillespie D (1997) An acoustic survey for sperm whales in the Southern Ocean sanctuary conducted from the RSV Aurora Australis. Rep Int Whal Comm 47:897–907

[19] Leaper R, Scheidat M (1998) An acoustic survey for cetaceans in the Southern Ocean Sanctuary conducted from the German government research vessel Polarstern. Rep Int Whal Comm 48:431–437

[20] Caldwell KD, Caldwell MC, Rice DW (1966) Behavior of the sperm whales, *Physeter catodon* In: Norris KS (ed) Whales, dolphins and porpoises. Unlv of Calif Press, Berkeley, pp 677–717

[21] Christal J, Whitehead H (1997) Aggregations of mature male sperm whales on the Galapagos Islands breeding ground. Mar Mamm Sci 13:59–69. 10.1111/j.1748-7692.1997.tb00612.x

[22] Lettevall E, Richter C, Jaquet N, Slooten E, Dawson S, Whitehead H, et al (2002) Social structure and residency in aggregations of male sperm whales. Can J Zool 80:1189–1196. 10.1139/z02-102

[23] Hammond PS, Mizroch SA, Donovan GP (eds) Individual recognition of cetaceans: use of photo-identification and other techniques to estimate population parameters. Rep Int Whal Comm. (Special Issue) 12:440

[24] Whitehead H, Gordon J (1986) Methods of obtaining data for assessing and modelling sperm whale populations which do not depend on catches. Reports of the International Whaling Commission (special issue) 8: 149–166.

[25] Arnbom T (1987) Individual identification of sperm whales. Rep Int Whal Comm 37:201–204

[26] Hinde RA (1976) Interactions, relationships and social structure. Man 1–17.

[27] Whitehead H (2008) Analyzing animal societies: quantitative methods for vertebrate social analysis. University of Chicago Press, Chicago, IL.

[28] Whitehead H, Dufault S (1999) Techniques for analyzing vertebrate social structure using identified individuals. Adv Stud Behav 28:33–74.

[29] Whitehead H (1995) Investigating structure and temporal scale in social organizations using identified individuals. Behav Ecol 6:199–208. 10.1093/beheco/6.2.199

[30] Whitehead H, Antunes R, Gero S, Wong SNP, Engelhaupt D, Rendell K (2012) Multilevel societies of female sperm whales (*Physeter macrocephalus*) in the Atlantic and Pacific: why are they so different? Int J Primatol 33:1142–1164. 10.1007/s10764-012-9598-z

[31] Cantor M, Whitehead H. 2015 How does social behavior differ among sperm whale clans? Mar Mamm Sci. 31:1275–1290. 10.1111/mms.12218

[32] Gero S, Gordon J, Whitehead H. (2015) Individualized social preferences and long-term social fidelity between social units of sperm whales. Anim Behav 102:15–23. 10.1016/j.anbehav.2015.01.008

[33] Christal J, Whitehead H (2001) Social affiliations within sperm whale (*Physeter macrocephalus*) groups. Ethology 107:323–340. 10.1046/j.1439-0310.2001.00666.x

[34] Whitehead H, Weilgart L (1991) Patterns of visually observable behavior and vocalizations in groups of female sperm whales. Behav 118:275–296. 10.1163/156853991X00328

[35] Whitehead H, Brennan S, Grover D (1992) Distribution and behaviour of male sperm whales on the Scotian Shelf, Canada. Can J Zool. 70:912–918. 10.1139/z92-130

[36] Konrad CM, Gero S, Frasier T, Whitehead H (2018) Kinship influences sperm whale social organization within, but generally not among, social units. R Soc Open Sci 5:180914 10.1098/rsos.180914 30225081 PMC6124104

[37] Madsen PT (2002) Sperm whale sound production -in the acoustic realm of the biggest nose on record. In Sperm whale sound production, Ph.D. dissertation, University of Aarhus, Denmark.

[38] Whitehead H (2009) SOCPROG programs: analysing animal social structures. Behav Ecol Sociobiol 63:765–778. 10.1007/s00265-008-0697-y

[39] Gero S, Gordon J, Whitehead H (2013) Calves as social hubs: dynamics of the social network within sperm whale units. Proc R Soc B 280:20131113 10.1098/rspb.2013.1113 23740785 PMC3774244

[40] Whitehead H (2007) Selection of models of lagged identification rates and lagged association rates using AIC and QAIC. Comm Statist Simulation Comput. 36:1233–1246. 10.1080/03610910701569531

[41] Whitehead H (2001) Analysis of animal movement using opportunistic individual identifications: application to sperm whales. Ecology 82:1417–1432

[42] Kobayashi H, Amano M (2020) Residency and abundance of sperm whales (*Physeter macrocephalus*) in Nemuro Strait, Hokkaido, Japan. Mar Mam Sci. 36:612–622. 10.1111/mms.12662

[43] Jaquet N, Dawson S, Slooten E (2000) Seasonal distribution and diving behavior of male sperm whales off Kaikoura: foraging implications. Can J Zool 78:407–403 10.1139/z99-208

[44] Wahlberg M (2002) The acoustic behaviour of diving sperm whales observed with a hydrophone array. J Exp Mar Biol Ecol 281:53–62. 10.1016/S0022-0981(02)00411-2

[45] Madsen PT, Møhl B (2000) Sperm whales (*Physeter catodon* L. 1758) do not react to sounds from detonators. J Acoust Soc Am 107:668–671. 10.1121/1.428568 10641677

[46] Gordon JCD (1987) Sperm whale groups and social bahvaiour observed off Sri Lanka. Rep Int Whal Comm 37:205–217

[47] Curé C, Antunes R, Alves AC, Visser F, Kvadsheim PH, Miller PJO (2013) Responses of male sperm whales (*Physeter macrocephalus*) to killer whale sounds: Implications for anti-predator strategies. Sci Rep 3:1579 10.1038/srep01579 23545484 PMC3613802

[48] Madsen PT, Payne R, Kristiansen NU, Wahlberg M, Kerr I, Mohl B (2002) Sperm whale sound production studied with ultrasound-time-depth-recording tags. J Exp Biol 205:1899–1906 12077166 10.1242/jeb.205.13.1899

[49] Best PB (1983) Sperm whale stock assessments and the relevance of historical whaling records. Rep Int Whal Comm. (Special Issue), 5:41–55.

[50] Kasuya T (1999) Examination of the reliability of catch statistics in the Japanese coastal sperm whale fishery. J Cetacean Res. Manag. 1:109–22

[51] Clutton-Brock TH (1989) Mammalian mating systems. Proc R Soc Lond B 236:339–372 10.1098/rspb.1989.0027 2567517

[52] Weilgart L, Whitehead H, Payne K (1996) A colossal convergence. Am. Sci. 84:278–287

[53] Evans KE, Harris S (2008) Adolescence in male African elephants, Loxodonta africana, and the importance of sociality. Anim. Behav. 76:779–787

[54] Chiyo PI, Archie EA, Hollister-Smith JA, Lee PC, Poole JH, Moss CJ, et al (2011) Association patterns of African elephants in all-male groups: the role of age and genetic relatedness. Anim Behav 81:1093–1099. 10.1016/j.anbehav.2011.02.013

[55] Wessel P, Smith WHF, Scharroo R, Luis R, Wobbe F (2013) Generic Mapping Tools: Improved Version Released, EOS Trans. AGU, 94(45): 409–410.

