# Correction: Long-term associations among male sperm whales (*Physeter macrocephalus*)

**DOI:** 10.1371/journal.pone.0262066

**Published:** 2021-12-30

**Authors:** The PLOS ONE Staff

## Notice of Republication

This article was republished on January 7, 2021, to correct errors in the figures. Please download this article again to view the correct version. The publisher apologizes for the errors.
