# Supplementary material for: Long-term associations among male sperm whales (Physeter macrocephalus)
Source: PLoS One. 2020 Dec 23;15(12):e0244204. doi: 10.1371/journal.pone.0244204 (PMC7757888; doi:10.1371/journal.pone.0244204)
Supplement: S1 Fig — Surveys were carried out from the Whale View Park in Rausu Town, Hokkaido, Japan (44°02'N, 145°13'E; 73 m above sea level) on 256 days from 2010 to 2019. Observation range is about 15 nmi, which substantially coincides with the range of photo-identification research. Four observers searched for whales using binoculars (Nikon MONARCH 12×42) and the position of each whale was recorded using digital theodolite (SOKKIA DT5). The distances between whales identified within 1 hour were calculated using the data collected during the 139 days for which the visibility was over 10 nmi and Beaufort Wind Scale was less than 5). (DOCX) [file pone.0244204.s001.docx]

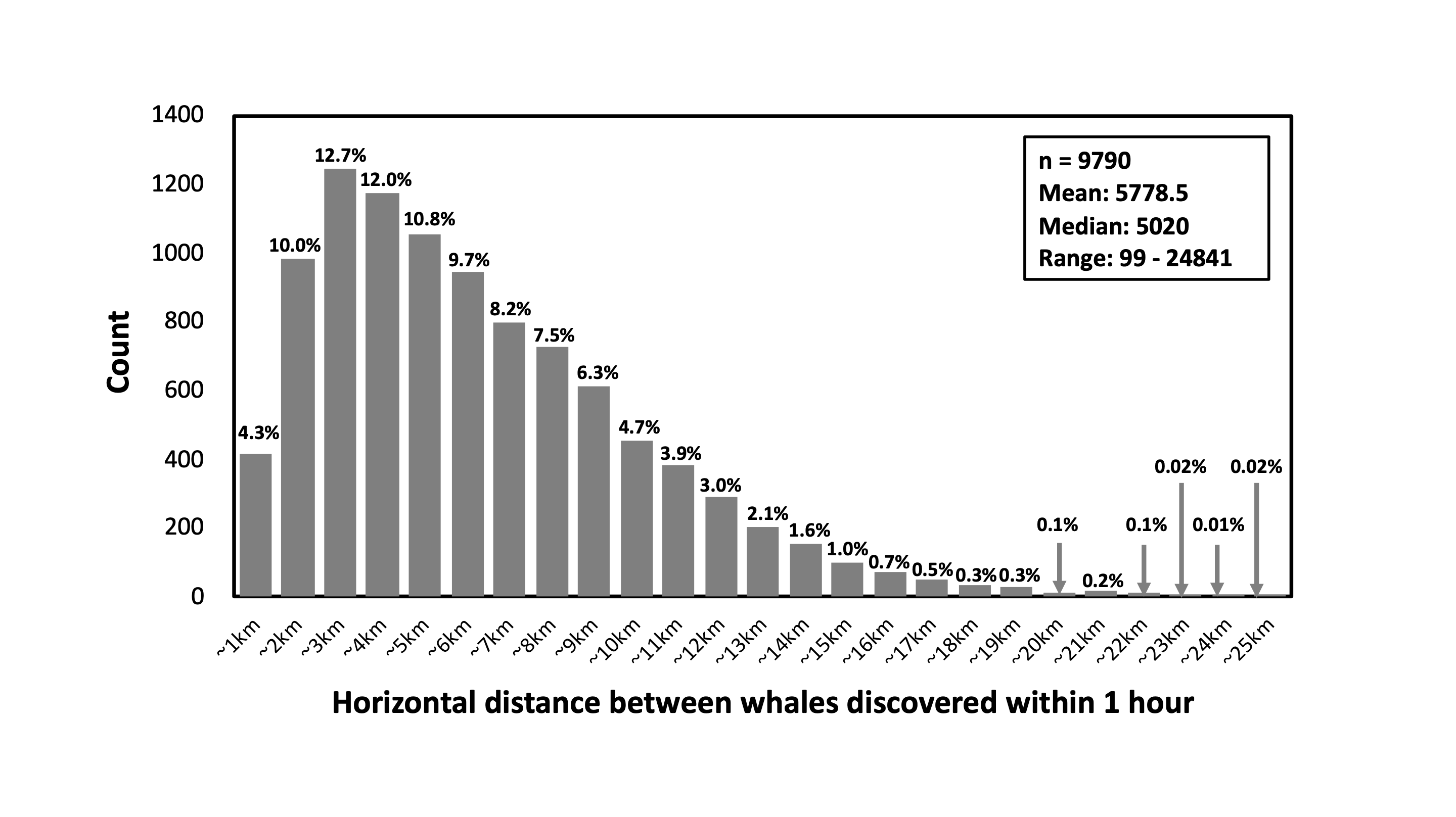


**S1 Fig. The distribution of horizontal distance between whales observed within 1 hour from land-based survey**

Surveys were carried out from the Whale View Park in Rausu Town, Hokkaido, Japan (44°02'N, 145°13'E; 73 m above sea level) on 256 days from 2010 to 2019. Observation range is about 15 nmi, which substantially coincides with the range of photo-identification research. Four observers search for whales using binoculars (Nikon MONARCH 12×42) and the position of each whale was recorded using digital theodolite (SOKKIA DT5). The distances between whales identified within 1 hour were calculated using the data collected during the 139 days for which the visibility was over 10 nmi and Beaufort Wind Scale was less than 5).
