# Supplementary material for: Long-term associations among male sperm whales (Physeter macrocephalus)
Source: PLoS One. 2020 Dec 23;15(12):e0244204. doi: 10.1371/journal.pone.0244204 (PMC7757888; doi:10.1371/journal.pone.0244204)
Supplement: S2 Fig — (DOCX) [file pone.0244204.s002.docx]

**S2 Fig. Discovery curves for individuals identified in Nermuro Strait between 2006 and 2017**
